# Diagnostic advantage of thin slice 2D MRI and multiplanar reconstruction of the knee joint using deep learning based denoising approach

**DOI:** 10.1038/s41598-022-14190-1

**Published:** 2022-06-20

**Authors:** Takahide Kakigi, Ryo Sakamoto, Hiroshi Tagawa, Shinichi Kuriyama, Yoshihito Goto, Masahito Nambu, Hajime Sagawa, Hitomi Numamoto, Kanae Kawai Miyake, Tsuneo Saga, Shuichi Matsuda, Yuji Nakamoto

**Affiliations:** 1 Department of Diagnostic Imaging and Nuclear Medicine, Kyoto University Graduate School of Medicine, 54 Shogoin Kawahara-cho, Sakyo-ku, Kyoto, 606-8507 Japan; 2 Preemptive Medicine and Lifestyle-Related Disease Research Center, Kyoto University Hospital, 53 Shogoin Kawahara-cho, Sakyo-ku, Kyoto, 606-8507 Japan; 3 Department of Orthopaedic Surgery, Kyoto University Graduate School of Medicine, 54 Shogoin Kawahara-cho, Sakyo-ku, Kyoto, 606-8507 Japan; 4 Department of Health Informatics, Kyoto University Graduate School of Medicine/School of Public Health, Yoshida Konoe-cho, Sakyo-ku, Kyoto, 606-8501 Japan; 5 MRI Systems Division, Canon Medical Systems Corporation, 1385 Shimoishigami, Otawara, Tochigi 324-8550 Japan; 6 Division of Clinical Radiology Service, Kyoto University Hospital, 54 Shogoin Kawahara-cho, Sakyo-ku, Kyoto, 606-8507 Japan; 7 Department of Advanced Medical Imaging Research, Kyoto University Graduate School of Medicine, 54 Shogoin Kawahara-cho, Sakyo-ku, Kyoto, 606-8507 Japan

**Keywords:** Trauma, Musculoskeletal system, Orthopaedics, Magnetic resonance imaging, Three-dimensional imaging

## Abstract

The purpose of this study is to evaluate whether thin-slice high-resolution 2D fat-suppressed proton density-weighted image of the knee joint using denoising approach with deep learning-based reconstruction (dDLR) with MPR is more useful than 3D FS-PD multi planar voxel image. Twelve patients who underwent MRI of the knee at 3T and 13 knees were enrolled. Denoising effect was quantitatively evaluated by comparing the coefficient of variation (CV) before and after dDLR. For the qualitative assessment, two radiologists evaluated image quality, artifacts, anatomical structures, and abnormal findings using a 5-point Likert scale between 2D and 3D. All of them were statistically analyzed. Gwet’s agreement coefficients were also calculated. For the scores of abnormal findings, we calculated the percentages of the cases with agreement with high confidence. The CV after dDLR was significantly lower than the one before dDLR (*p* < 0.05). As for image quality, artifacts and anatomical structure, no significant differences were found except for flow artifact (*p* < 0.05). The agreement was significantly higher in 2D than in 3D in abnormal findings (*p* < 0.05). In abnormal findings, the percentage with high confidence was higher in 2D than in 3D (*p* < 0.05). By applying dDLR to 2D, almost equivalent image quality to 3D could be obtained. Furthermore, abnormal findings could be depicted with greater confidence and consistency, indicating that 2D with dDLR can be a promising imaging method for the knee joint disease evaluation.

## Introduction

MRI of the knee joint is widely used to evaluate bone, cartilage, meniscus and ligament injuries. Although arthroscopy is the gold standard, MRI is necessary for diagnosis, treatment and preoperative evaluation^1^.

In two-dimensional (2D) fast spin echo (FSE) sequences, multiple cross-sections, such as coronal, sagittal, and transverse images, should be scanned independently. These sequences are characterized by high tissue contrast and have the advantage of high in-plane spatial resolution. However, the disadvantages are relatively thick slices and partial volume effect^2,3^. For three-dimensional (3D) FSE sequences such as multi-planar voxel (MPV, Canon Medical Systems Corporation) and sampling perfection with application optimized contrast using different flip angle evolutions (SPACE, Siemens Healthineers), the MR signal can be acquired by volume excitation instead of single slice excitation in 2D sequences, which results in higher signal and thinner slice thicknesses through the use of z-direction phase encoding. Because 3D FSE sequences collect data with a small isotropic voxel size, it can provide MPR (multiplanar reconstruction) images and make arbitrary cross-sectional images. Additionally, it has less partial volume effect than ordinary 2D FSE sequences^2–4^. However, because of blurring and low resolution, earlier reports have described that the depiction of meniscus injuries is inferior to that of 2D FSE sequences^5,6^.

In clinical practice, MR images should be scanned within an acceptable time, and there are several ways to obtain high resolution images under such situations. The super-resolution technique is one of them. This is a technique that transforms low resolution images into high resolution images. Recent reports show a super-resolution technique using multi-contrast MR images, which made it possible to obtain high resolution image from under-sampled image acquired in a short scan time using auxiliary information from a fully-sampled sequence^7,8^. Another technique is to obtain high resolution images with a short scan time and then denoise. Recently, many denoising techniques based on a deep learning algorithm for MRI have been reported^9–12^. One of these techniques involves training a neural network to perform computational generation of images that closely resemble high-quality training images from input images that include large amounts of noise, leading to the creation of a deep convolutional neural network (DCNN)^9,10^. By installing the DCNN in a diagnostic imaging system, low signal-to-noise ratio (SNR) images acquired by the system can be denoised and can be converted to high SNR images^9,10,13^. Advanced intelligent Clear-IQ Engine (AiCE, Canon Medical Systems Corporation) is a state of art denoising technique with deep learning reconstruction (dDLR) and has previously been reported to be applied to various sites, including the pelvic region and liver^14–20^. dDLR makes it possible to obtain low-noise thin-slice 2D MR images within an acceptable scan time. Using this technique, we can obtain almost isotropic voxel data in a high-resolution 2D manner and create MPR images. Such images with both high in-plane resolution with less blurring and a multi-planar view can be ideal for knee joint image.

This study was designed to evaluate whether thin-slice 2D FS-PDWI of the knee joint after dDLR with MPR was more useful than 3D FS-PD MPV image.

## Materials and methods

### Study design

This prospective study was institutional review board (Ethics committee of Kyoto university graduate school and faculty of medicine)-approved and was registered with the UMIN Clinical Trials Registry (UMIN000036700). It was performed in accordance with the ethical standards as laid down in the 1964 Declaration of Helsinki and its later amendments. Written informed consent was obtained from all individual participants included in the study. The primary end points were to assess the denoising effects of dDLR on thin-slice 2D FS-PDWI. The secondary end points were evaluation of the performance of dDLR-applied thin-slice 2D FS-PDWI of the knee joint with MPR images in comparison with 3D FS-PD MPV image in terms of the image quality, artifacts, the visualization of anatomical structures, and the detection of abnormal findings. This study did not require direct arthroscopic observation as a validated reference standard.

### Subjects

We recruited patients who were going to receive MRIs for knee problems between January and March 2020. Inclusion criteria were that they were at least 20 years of age and agreed to participate in our study. Exclusion criteria included general contraindications for MRI, prior knee surgery and the use of different coils for body size.

### MR image acquisition

MR scans were conducted using a 3T scanner (Vantage Galan 3T/ZGO, Canon Medical Systems, Tochigi, Japan) with a 16-channel knee coil. As our institutional protocol for routine knee MRI, sagittal and coronal proton density-weighted (repetition time msec/echo time msec, 4326/20; matrix, 384 × 448; 3 mm thickness), sagittal and coronal T2-weighted (repetition time msec/echo time msec, 5600/80.5; matrix, 320 × 448; 3 mm thickness), coronal STIR (repetition time msec/echo time msec, 4443/60; inversion time, 200 ms; matrix, 320 × 448; 3 mm thickness) and sagittal 3D FS-PD MPV images (voxel dimension, 0.7 × 0.7 × 0.7 mm) were acquired. Additionally, we performed coronal thin-slice 2D FS-PDWI (resolution, 0.5 × 0.5 mm; 1 mm thickness; − 0.3 mm gap) (Table 1).

**Table 1:** MR pulse sequence protocol.

Sequences	2D FS-PDWI	3D FS-PD MPV Image
Orientation	Coronal	Sagittal
Repetition time, ms	12,400	600
Echo time, ms	10	48.8
Acceleration factor	2	2
Echo train length	7	25
Receiver bandwidth, Hz/pixel	195	488
Flip/flop angle, degree	90/180	90/170 (Variable)
Field of view, mm	160 × 160	170 × 180
Matrix	320 × 320	240 × 256
Slice thickness/gap, mm	1/− 0.3	0.7/0
Voxel dimension, mm	0.5 × 0.5 × 0.7	0.7 × 0.7 × 0.7
Voxel volume, mm^3^	0.175	0.343
Number of excitations	1	1
Number of slices	128	160
Concatenations	1	1
Phase encoding direction	Head to foot	Anterior to posterior
Phase sampling, %	53.8	59
Acquisition time	4 min, 58 s	5 min, 50 s

*MR* indicates magnetic resonance, *2D* 2-dimensional, *FS-PDWI* fat saturated-proton density weighted image, *3D* 3-dimensional, *FS-PD MPV* fat saturated-proton density multi planar voxel.

### dDLR application to MRI data

dDLR, a denoising technique used as a product (AiCE, Canon Medical Systems Corporation), was adapted to the scanned coronal thin-slice 2D FS-PDWI in this study.

An MRI denoising method based on the denoising convolutional neural network (CNN) approach has been reported: shrinkage convolutional neural network (SCNN)^9^. Both the denoising CNN and SCNN have residual learning and batch-normalization in the hidden layer. Unlike the denoising CNN, SCNN can adjust the noise power of the input image using a CNN with soft-shrinkage activation functions. SCNN can adapt to various noise levels in a single network by setting an appropriate noise level for each input image. This means that there is no need to train a separate CNN for each noise level. dDLR is based on SCNN, but differs in its denoising technique. While SCNN performs noise reduction directly in the image domain, dDLR performs noise reduction by learning the noise threshold of the high-frequency components extracted by a discrete cosine transform (DCT)^9,10,15,16^.

### MPR image

Sagittal and transverse images were created from coronal thin-slice 2D FS-PDWI on the MRI console (slice thickness, 1 mm; slice spacing, 1 mm). Similarly, coronal and transverse images were created from the 3D FS-PD MPV sagittal image (slice thickness, 0.7 mm; slice spacing, 0.7 mm).

### Quantitative image evaluation methods

To evaluate the image noise before and after the adaptation of dDLR, two board-certified radiologists with 15 years of experience (R.S. and T.K.) placed the region of interests (ROIs) on the femur, the pars intermedia of the lateral meniscus, and the ACL on a medical professional imaging viewer (EV Insite, PSP Corporation, Tokyo, Japan) and obtained the mean, standard deviation (SD), and coefficient of variation (CV, SD divided by mean) of the signal in coronal thin-slice 2D FS-PDWI before and after the adaptation of dDLR. The sizes of the ROI were 50 mm^2^, 4 mm^2^, and 5 mm^2^.

### Qualitative image evaluation methods

Corresponding thin-slice 2D FS-PDWI and 3D FS-PD MPV image datasets were anonymized and randomized. Each study was evaluated independently by two board-certified radiologists with 9 years and 15 years of experience (H.T. and T.K.). Both readers used medical professional imaging viewer (Centricity Universal Viewer, GE Healthcare, Chicago, IL). Before the evaluation, the readers mutually discussed the evaluation method and agreement to proceed. Reading was performed at an approximately 1 lx using 21.3-inch diagnostic-quality color liquid crystal display monitor that was calibrated to Digital Imaging and Communications in Medicine standards (RadiForce RS240, EIZO Corporation, Ishikawa, Japan). All data were displayed in a 2 × 2 layout on the display monitor. Both readers were allowed to set the windows and levels as desired, as well as to magnify and scroll freely. Both thin-slice 2D FS-PDWI and 3D FS-PD MPV image can be viewed using interactive MPR mode.

The evaluation items included image quality assessment, artifacts and anatomical structure visualization. All of them were evaluated on a 5-point Likert scale. Abnormal findings were also evaluated using a 5-point Likert scale^21–23^.

For image quality, we evaluated edge sharpness, contrast resolution, and fluid brightness among coronal, sagittal and transverse images. We also evaluated uniformity of image quality by comprehensively assessing the three image planes. A 5-point Likert scale was used as the evaluation criterion: 1 point, very bad; 2 points, bad; 3 points, sufficient; 4 points, good; 5 points, very good.

With regard to artifacts, noise, motion, flow and aliasing artifacts were evaluated by comprehensively assessing the three image planes using a 5-point Likert scale: 1 point, severe—difficult to assess for diagnosis; 2 points, moderate—remarkable artifacts are present; 3 points, mild—artifacts are seen, but not so readily apparent; 4 points, minimal—few artifacts are visible; 5 points, none. Aliasing artifacts were evaluated as not particularly problematic as long as they did not overlay the structures being evaluated.

Delineation of the following anatomical structures was evaluated. Femur (coronal, sagittal, and transverse images); medial and lateral meniscus (coronal, sagittal, and transverse images); articular cartilage (tibiofemoral and femoropatellar joints) (tibiofemoral joint on coronal and sagittal images & femoropatellar joints on sagittal and transverse images); anterior cruciate and posterior cruciate ligaments (ACL and PCL) (coronal, sagittal, and transverse images); and medial and lateral collateral ligaments (coronal images). A 5-point Likert scale was used as the evaluation criterion: 1 point, very poor—the anatomical structure is completely obscured; 2 points, bad—some anatomical structures are unclear; 3 points, sufficient—anatomical details are not sufficiently clear; 4 points, good—anatomical structures are mostly clear at a minimum of detail; 5 points, very good—details of all anatomical structures are clear.

Depiction of the following abnormal findings for each structure were evaluated. 1. Femur (coronal, sagittal, and transverse images): Assessment of osteochondral lesions related to arthritis including bone marrow edema-like lesion and subchondral cyst-like lesions. Bone marrow edema-like lesion was defined as a non-cystic subchondral area of ill-defined hyperintensity on fluid-sensitive sequences. Subchondral cyst-like lesions were well defined rounded areas of fluid signal intensity^24^. 2. Medial and lateral meniscus (coronal, sagittal, and transverse images): Only tears were evaluated. A tear was defined as meniscal distortion or increased intrasubstance signal intensity unequivocally contacting the articular surface^25^. 3. Articular cartilage (tibiofemoral and femoropatellar joints) (coronal, sagittal, and transverse images): Cartilage injury was evaluated and defined based on the Noyes classification system^26,27^. 4. ACL and PCL (coronal, sagittal, and transverse images): Complete and partial tears were evaluated. Complete ACL and PCL tears were defined as complete discontinuity of fibers or irregular contour with increased signal intensity. Partial ACL tear was defined as abnormal intra-ligament signal, bowing of the ligament, and inability to identify all fibers. Partial PCL tear was defined as hyperintense signal alterations without complete disruption of the ligament^28,29^. Also, ACL with ganglion cysts was evaluated and was defined as cysts that were either on the surface or within the substance of the ligament and showed water signal^30–33^. 5. Medial collateral ligaments (MCL) (superficial and deep layers) and lateral collateral ligaments (LCL) (coronal image): Injury and tear were evaluated and defined based on the grading system^29,34^. The images specified above were viewed and evaluated overall. The evaluation criteria were used with the following confidence levels: 1 point, certainly absent; 2 points, probably absent; 3 points, equivocal; 4 points, probably present; 5 points, certainly present^35^. If no abnormal signal intensity was found in the anatomical structures above, they were considered normal.

### Statistical analysis

Statistical analyses were performed using JMP Pro 15.2.0 software (SAS Institute, Cary, NC). For the quantitative evaluation, intraclass correlation coefficients (ICCs) of CV by the two radiologists were obtained. The mean values of CV by the two radiologists before and after the adaptation of dDLR were compared using the Mann–Whitney U test.

Inter-rater reliability coefficients between thin-slice 2D FS-PDWI and 3D FS-PD MPV image were calculated using Gwet’s AC_2,_ and compared using the Mann–Whitney U test for abnormal findings. Calculation of Gwet's AC_2_ was performed with R version 4.1.0 using irrCAC package version 1.0 (https://CRAN.R-project.org/package=irrCAC). Agreement level was defined as follows: less than 0, poor; 0–0.2, slight; 0.21–0.4, fair; 0.41–0.6, moderate; 0.61–0.8, substantial; and 0.81–1, almost-perfect agreement^36^. In addition, 95% confidence intervals (CIs) were calculated.

For the evaluation of image quality, artifacts, and anatomical structure visualization, scores of 3, 4, and 5 were classified as "acceptable" and scores of 1 and 2 as "non-acceptable". For this dichotomous classification, the results were statistically analyzed by chi-squared test.

We defined "abnormal findings" as follows: "on both thin-slice 2D FS-PDWI and 3D FS-PD MPV images", " on two or more image planes (coronal image only for medial and lateral collateral ligament)", and “scored 4 or higher by two readers”. For "abnormal findings", we calculated the percentages of both scores of 5 (5/5) for the number of image plane in the evaluated structures and analyzed with chi-squared test. *P* values of 0.05 and less were considered statistically significant.

## Results

Thirteen patients (4 males and 9 females; mean age 64.5 years old, range from 35 to 87 years old) who received MRI of the knee joint were enrolled between January and March 2020. Fourteen knees (3 right knees and 11 left knees) were examined because both knees were examined in one patient. We excluded one patient, who was examined using different coil because of knee size issue, from the evaluation. Therefore, MRI of thirteen knees (2 right knees and 11 left knees) were evaluated.

**Figure 1:**
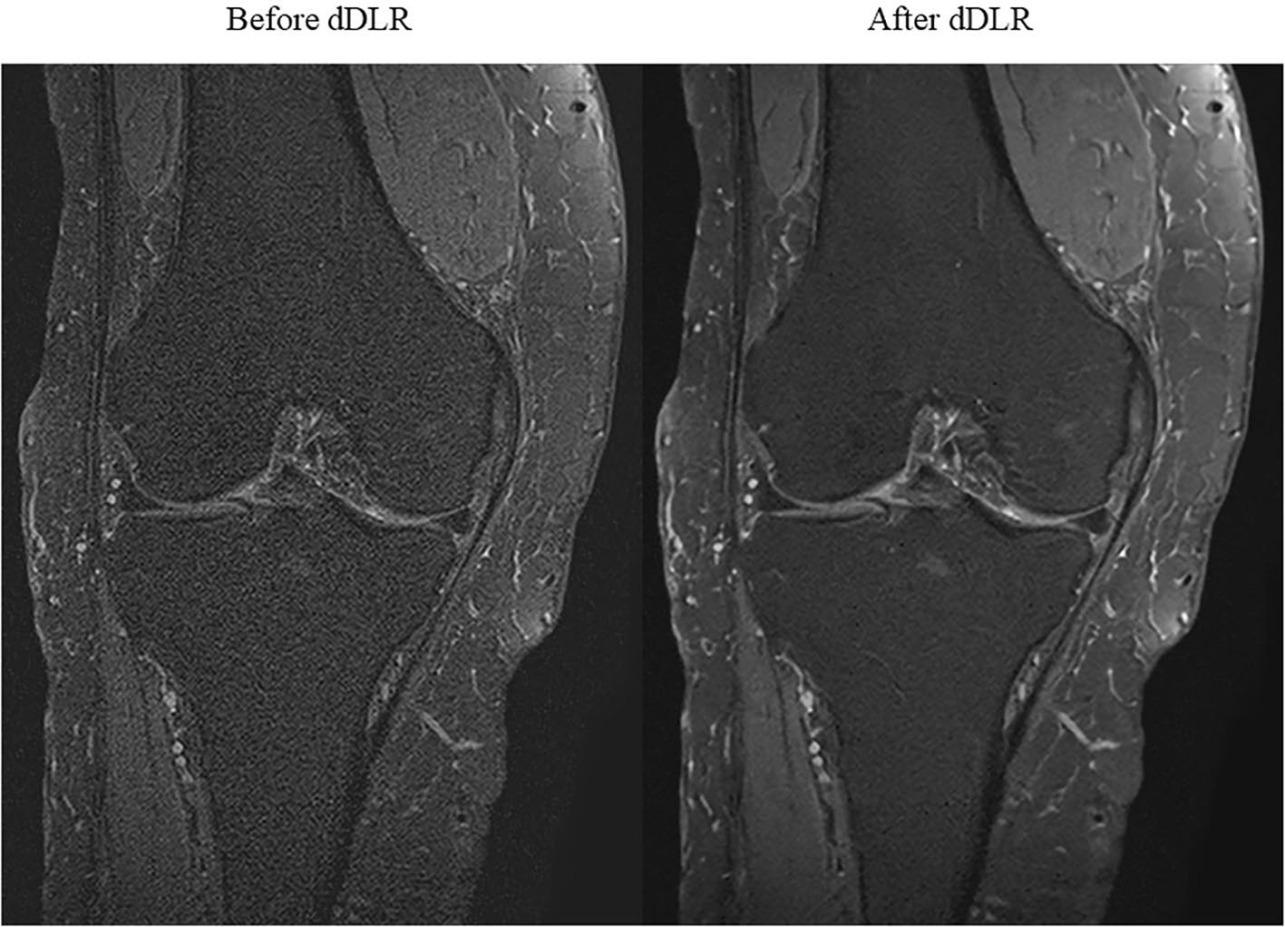
Thin-slice 2D FS-PDWI in a 73-year-old woman before and after the adaptation of dDLR. Images after dDLR are denoised, facilitating observation of the anatomical structures. 2D FS-PDWI indicates 2-dimensional fat saturated-proton density weighted image; dDLR, denoising approach with deep learning-based reconstruction.

**Figure 2:**
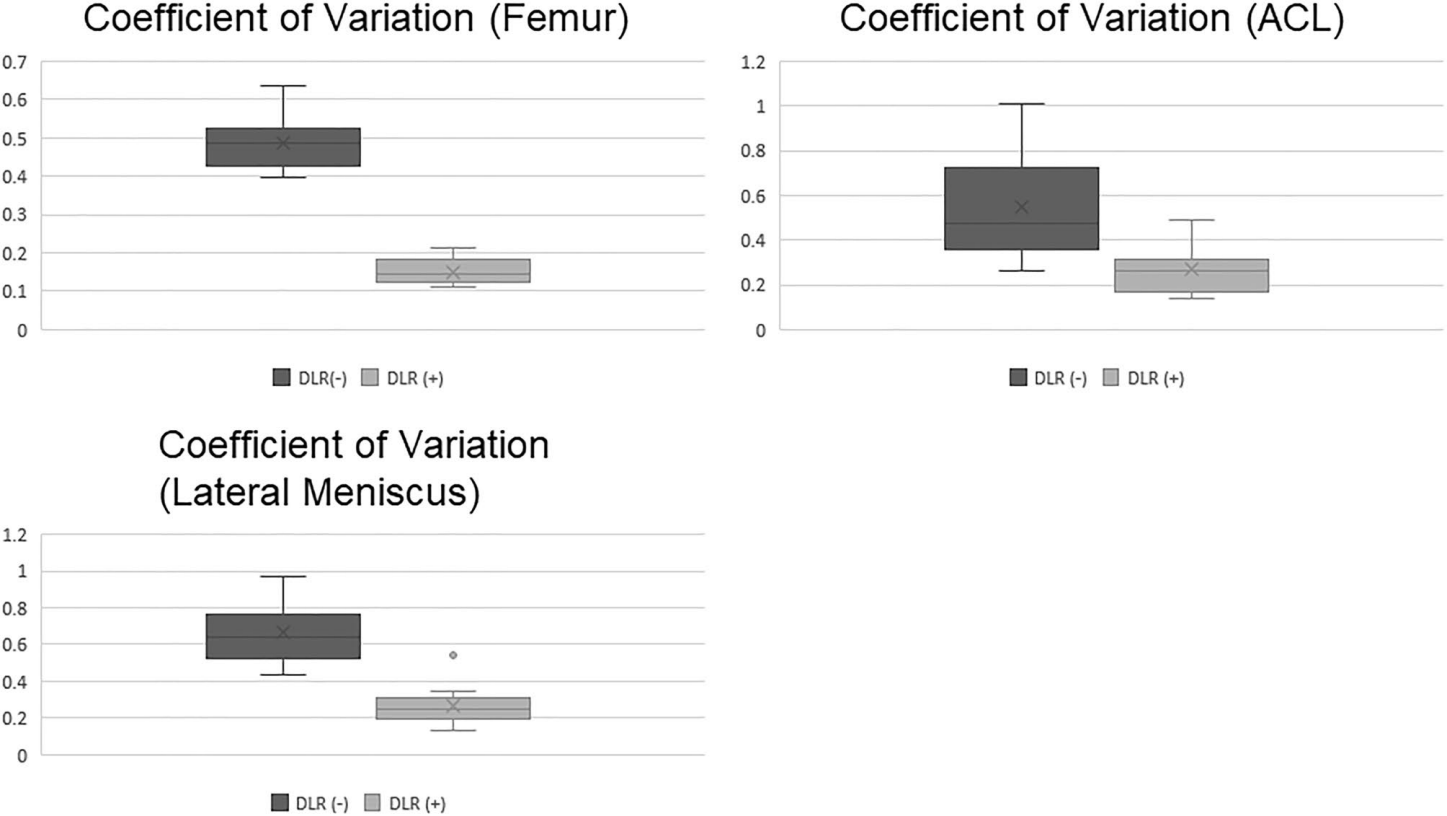
Results of CV measurement of the femur, the pars intermedia of the lateral meniscus and ACL in coronal thin-slice 2D FS-PDWI before and after the adaptation of dDLR. The CV after dDLR was significantly lower than that before dDLR at all sites (*p* < 0.05, *p* < 0.05, and *p* < 0.05, respectively). CV indicates coefficient of variation; ACL, anterior cruciate ligament; 2D FS-PDWI, 2-dimensional fat saturated-proton density weighted image; dDLR, denoising approach with deep learning-based reconstruction.

As the results of quantitative image evaluation methods, ICCs of CV measured by two radiologists were 0.82–0.96 for all ROI sizes, which were recognized as excellent interobserver reproducibility^37^. The CV after dDLR was significantly lower than that before dDLR for the femur, lateral meniscus, and ACL (*p* < 0.05, *p* < 0.05, and *p* < 0.05, respectively) (Figs. 1 and 2).

**Figure 3:**
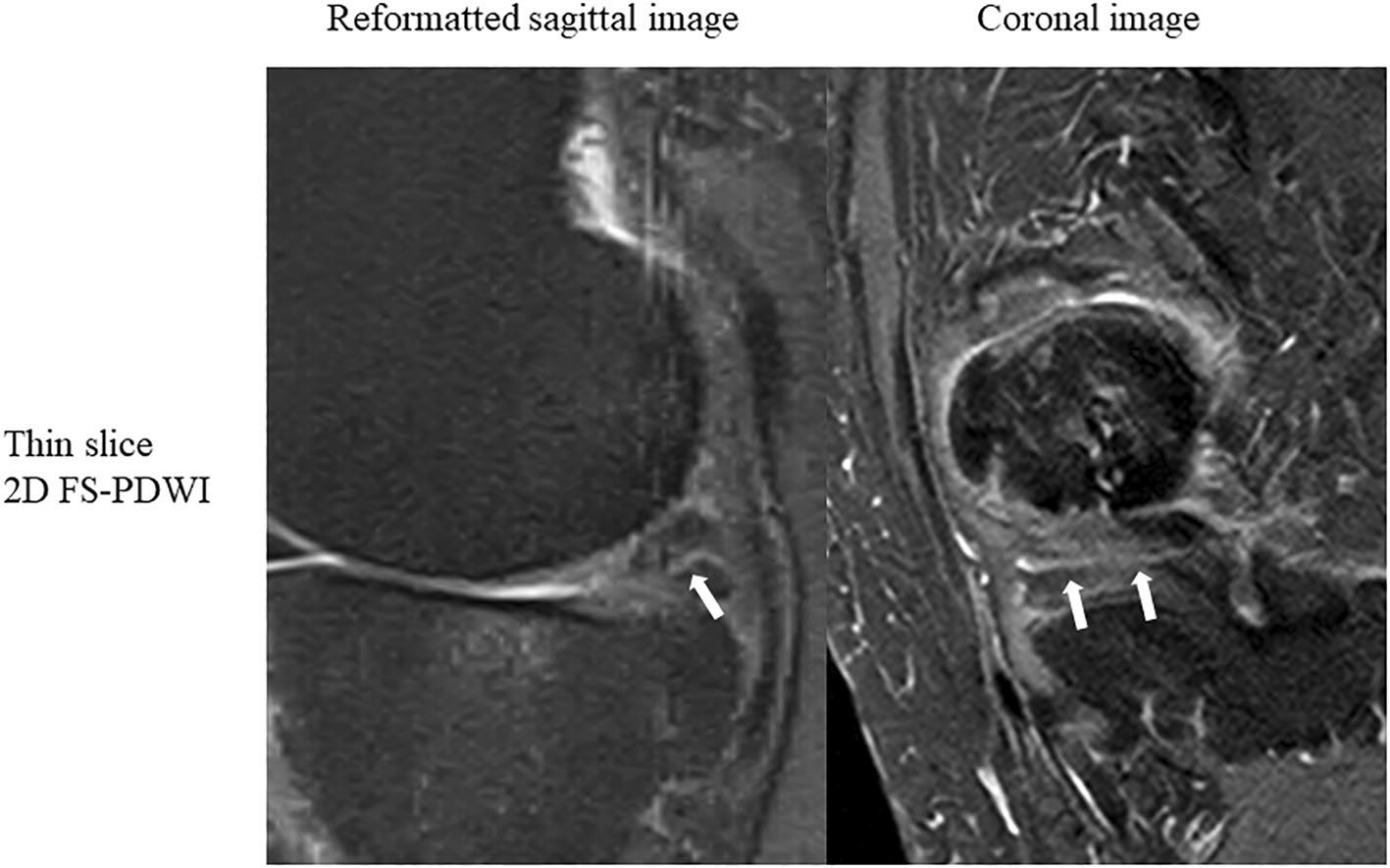
A 58-year-old woman with horizontal tear of the posterior horn of the medial meniscus (arrows). The flow artifact of the popliteal artery on sagittal image covers the posterior horn, making it slightly difficult to see, but the horizontal tear is clearly delineated. 2D FS-PDWI indicates 2-dimensional fat saturated-proton density weighted image.

Results of inter-rater reliability coefficients show that image quality, artifacts, and delineation of anatomical structures in thin-slice 2D FS-PDWI and 3D FS-PD MPV images produced excellent agreement between the two radiologists, with Gwet’s AC_2_ value of 0.99 (95% CI: 0.98, 0.99) & 0.99 (95% CI: 0.98, 0.99), 0.99 (95% CI: 0.99, 1) & 0.99 (95% CI: 0.99, 1), and 0.98 (95% CI: 0.98, 0.99) & 0.98 (95% CI: 0.98, 0.99), respectively. For dichotomous evaluation of image quality and artifacts, the only significantly different parameter evaluated were those related to flow artifacts between two radiologists (*p* < 0.05) (Fig. 3). Otherwise, no other significant differences were found (Table 2). No significant difference was found in the evaluation of anatomical structure visualization (Table 3).

**Table 2:** Evaluation of image quality and artifacts of thin-slice 2D FS-PDWI and 3D FS-PD MPV image by two readers.

		Reader	Evaluation (2D/3D)	
			Acceptable	Non-acceptable
Edge sharpness	Coronal	A	13/13	0/0
		B	13/13	0/0
	Sagittal	A	12/13	1/0
		B	13/13	0/0
	Transverse	A	12/13	1/0
		B	13/13	0/0
Contrast resolution	Coronal	A	13/13	0/0
		B	13/13	0/0
	Sagittal	A	13/13	0/0
		B	13/13	0/0
	Transverse	A	13/13	0/0
		B	13/13	0/0
Fluid brightness	Coronal	A	13/13	0/0
		B	13/13	0/0
	Sagittal	A	13/13	0/0
		B	13/13	0/0
	Transverse	A	13/13	0/0
		B	13/13	0/0
Uniformity of image**		A	12/13	1/0
		B	13/13	0/0
Artifacts**	Noise	A	13/13	0/0
		B	13/13	0/0
	Motion	A	11/13	2/0
		B	12/13	1/0
	Flow	A*	0/13	13/0
		B*	2/13	11/0
	Aliasing	A	13/13	0/0
		B	13/13	0/0

Acceptable, scores 3, 4 and 5; Non-acceptable, scores 1 and 2.

Coronal and sagittal images were scanned as original images in thin-slice 2D FS-PDWI and 3D FS-PD MPV image, respectively. Others were MPR images.

2D FS-PDWI indicates 2-dimensional fat saturated-proton density weighted image; 3D FS-PD MPV, 3-dimensional fat saturated-proton density multi planar voxel.

*p value < 0.05 by chi-squared test.

** “Uniformity of image” and “Artifacts” were evaluated by referring all three planes.

**Table 3:** Evaluation of anatomical structure visualization of thin-slice 2D FS-PDWI and 3D FS-PD MPV image by two readers.

		Reader	Evaluation (2D/3D)	
			Acceptable	Non-acceptable
Femur	Coronal	A	13/13	0/0
		B	13/13	0/0
	Sagittal	A	12/13	1/0
		B	13/13	0/0
	Transverse	A	13/13	0/0
		B	13/13	0/0
Medial meniscus	Coronal	A	13/13	0/0
		B	13/13	0/0
	Sagittal	A	13/13	0/0
		B	13/13	0/0
	Transverse	A	13/13	0/0
		B	13/13	0/0
Lateral meniscus	Coronal	A	13/13	0/0
		B	13/13	0/0
	Sagittal	A	13/13	0/0
		B	13/13	0/0
	Transverse	A	13/13	0/0
		B	13/13	0/0
Tibiofemoral joints	Coronal	A	13/13	0/0
		B	13/13	0/0
	Sagittal	A	13/13	0/0
		B	13/13	0/0
Femoropatellar joints	Sagittal	A	12/13	1/0
		B	13/13	0/0
	Transverse	A	13/13	0/0
		B	13/13	0/0
Anterior cruciate ligament	Coronal	A	13/13	0/0
		B	13/13	0/0
	Sagittal	A	12/13	1/0
		B	13/13	0/0
	Transverse	A	13/13	0/0
		B	13/13	0/0
Posterior cruciate ligament	Coronal	A	13/13	0/0
		B	13/13	0/0
	Sagittal	A	12/13	1/0
		B	13/13	0/0
	Transverse	A	13/13	0/0
		B	13/13	0/0
Medial collateral ligament	Coronal	A	13/13	0/0
		B	13/13	0/0
Lateral collateral ligament	Coronal	A	13/13	0/0
		B	13/13	0/0

Acceptable, scores 3, 4 and 5; Non-acceptable, scores 1 and 2.

Coronal and sagittal images were scanned as original images in thin-slice 2D FS-PDWI and 3D FS-PD MPV image, respectively. Others were MPR images.

2D FS-PDWI indicates 2-dimensional fat saturated-proton density weighted image; 3D FS-PD MPV, 3-dimensional fat saturated-proton density multi planar voxel.

*p value < 0.05 by chi-squared test.

The results of inter-rater reliability coefficients for abnormal findings were shown in Table 4. Statistically, the inter-rater reliability coefficients in abnormal findings, which were analyzed by adding all image planes, were significantly higher in thin-slice 2D FS-PDWI than in 3D FS-PD MPV image (*p* < 0.05).

**Table 4:** Inter-rater reliability coefficients using Gwet’s AC_2_ in abnormal findings.

Abnormal findings	2D FS-PDWI*				3D FS-PD MPV image			
Femur	Overall	0.87 (0.72, 1)	Coronal	0.84 (0.52, 1)	Overall	0.88 (0.77, 0.99)	Coronal	0.86 (0.63, 1)
			Sagittal	0.82 (0.43, 1)			Sagittal	0.84 (0.59, 1)
			Transverse	0.97 (0.93, 1)			Transverse	0.93 (0.81, 1)
Medial meniscus	Overall	0.97 (0.94, 1)	Coronal	0.99 (0.98, 1)	Overall	0.94 (0.89, 0.99)	Coronal	0.98 (0.94, 1)
			Sagittal	0.96 (0.87, 1)			Sagittal	0.89 (0.75, 1)
			Transverse	0.95 (0.87, 1)			Transverse	0.95 (0.87, 1)
Lateral meniscus	Overall	0.96 (0.92, 1)	Coronal	0.95 (0.86, 1)	Overall	0.92 (0.86, 0.98)	Coronal	0.96 (0.87, 1)
			Sagittal	0.96 (0.88, 1)			Sagittal	0.89 (0.75, 1)
			Transverse	0.97 (0.90, 1)			Transverse	0.92 (0.8, 1)
Tibiofemoral joints	Overall	0.99 (0.98, 1)	Coronal	0.99 (0.98, 1)	Overall	0.96 (0.91, 1)	Coronal	0.97 (0.93, 1)
			Sagittal	0.99 (0.98, 1)			Sagittal	0.95 (0.86, 1)
Femoropatellar joints	Overall	0.99 (0.98, 1)	Sagittal	0.99 (0.98, 1)	Overall	0.62 (0.36, 0.88)	Sagittal	0.59 (0.18, 1)
			Transverse	0.99 (0.98, 1)			Transverse	0.65 (0.27, 1)
Anterior cruciate ligament	Overall	0.93 (0.89, 0.98)	Coronal	0.93 (0.85, 1)	Overall	0.92 (0.88, 0.97)	Coronal	0.93 (0.85, 1)
			Sagittal	0.93 (0.85, 1)			Sagittal	0.93 (0.86, 1)
			Transverse	0.93 (0.85, 1)			Transverse	0.92 (0.83, 1)
Posterior cruciate ligament	Overall	0.99 (0.99, 1)	Coronal	0.99 (0.97, 1)	Overall	0.96 (0.94, 0.99)	Coronal	0.96 (0.92, 1)
			Sagittal	0.99 (0.98, 1)			Sagittal	0.97 (0.94, 1)
			Transverse	0.99 (0.98, 1)			Transverse	0.96 (0.92, 1)
Medial collateral ligament			Coronal	0.84 (0.66, 1)			Coronal	0.90 (0.79, 1)
Lateral collateral ligament			Coronal	0.98 (0.95, 1)			Coronal	0.98 (0.95, 1)

2D FS-PDWI indicates 2-dimensional fat saturated-proton density weighted image; 3D FS-PD MPV, 3-dimensional fat saturated-proton density multi planar voxel.

Numbers in parenthesis are 95% confidence intervals (95% CI).

*p value < 0.05 by the Mann–Whitney U test, when analyzed by adding all image planes.

**Figure 4:**
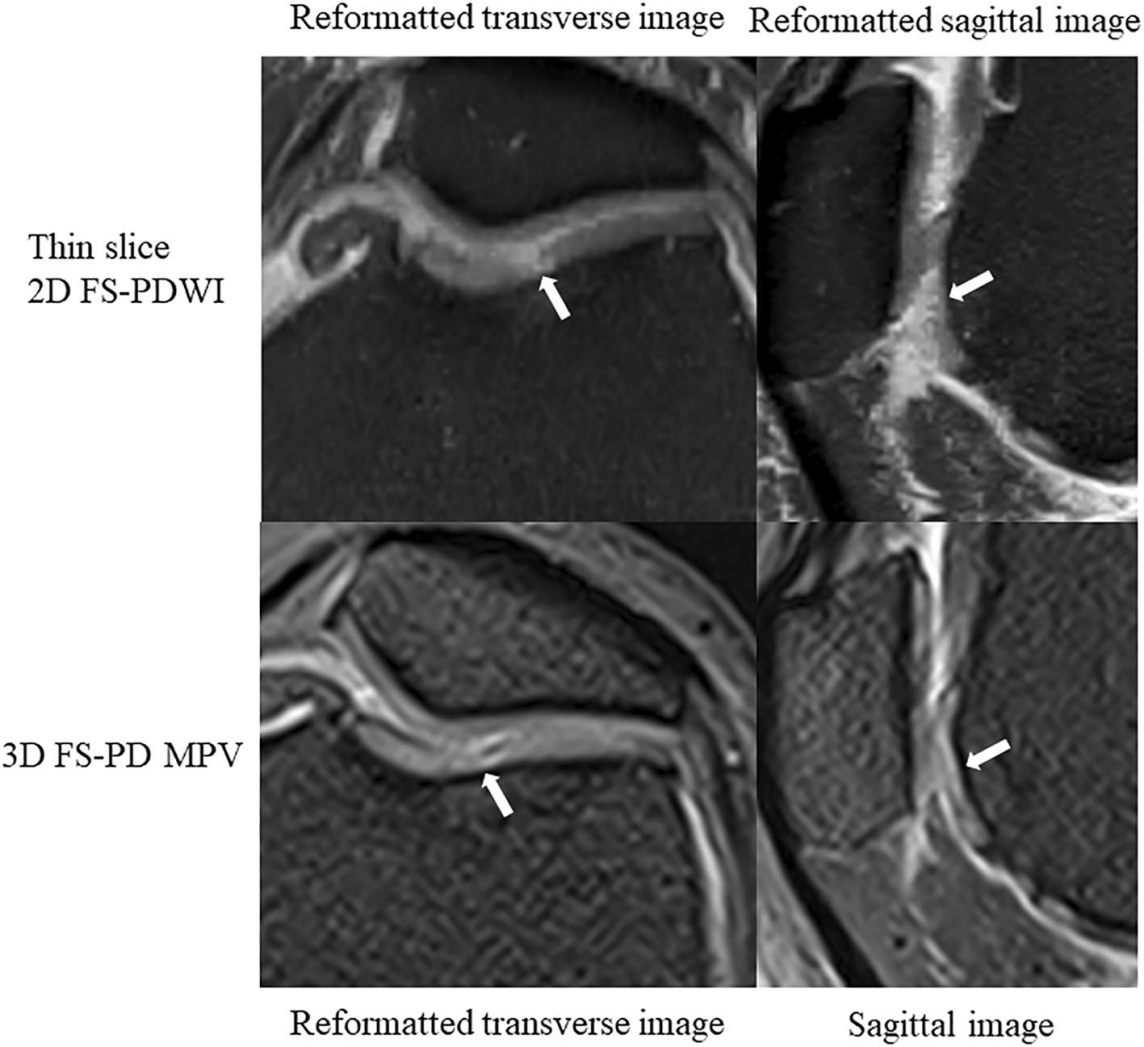
A 58-year-old woman with chondral defect of the femoral trochlea (arrow). Thin-slice 2D FS-PDWI shows the chondral defect more clearly than 3D FS-PD MPV image, whereas 3D FS-PD MPV image shows high signal intensity. This is because the defective area is obscured by blurring. 2D FS-PDWI indicates 2-dimensional fat saturated-proton density weighted image; 3D FS-PD MPV, 3-dimensional fat saturated-proton density multi planar voxel.

**Figure 5:**
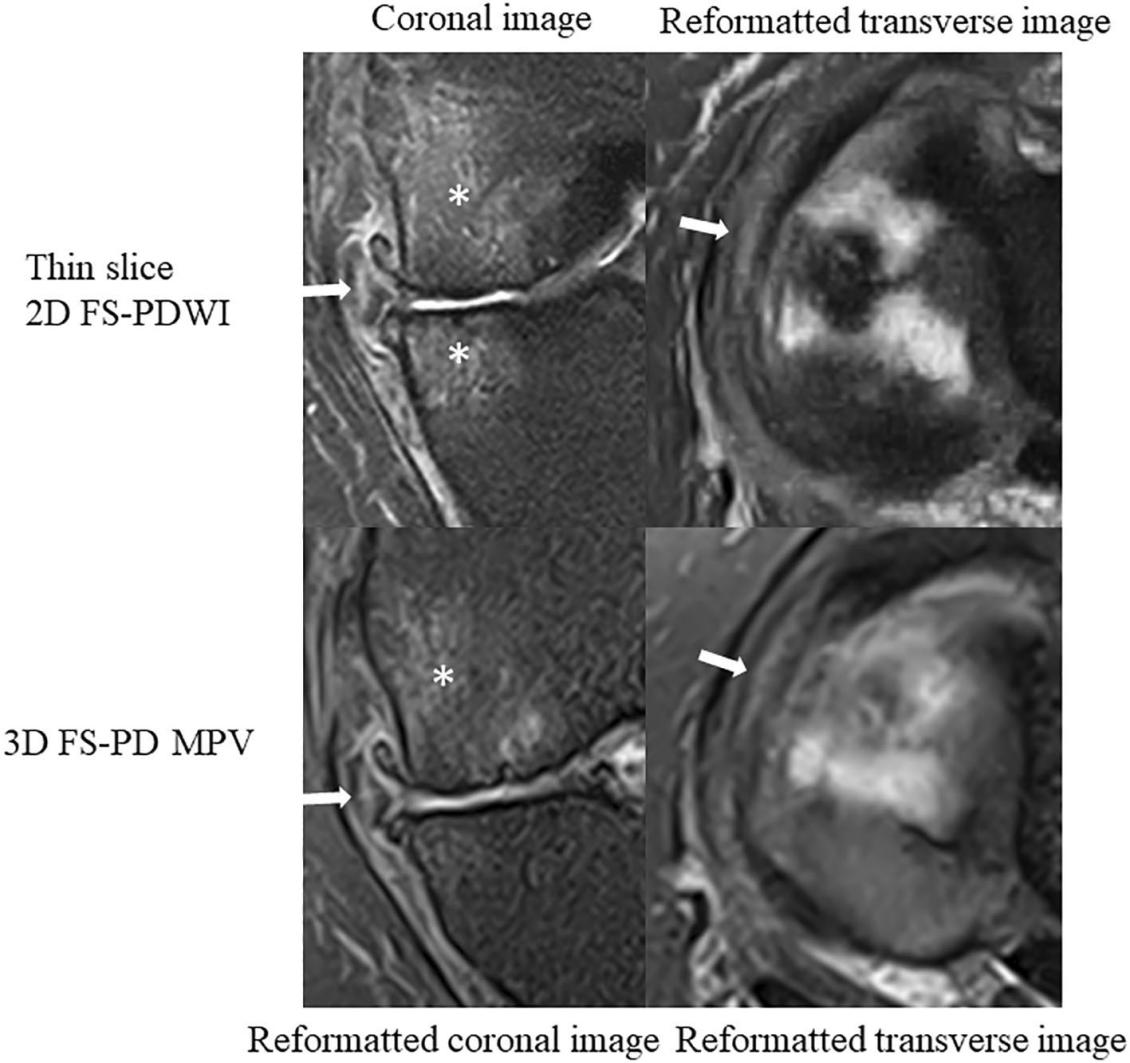
A 58-year-old woman with vertical tear of the medial meniscus (arrow) and bone marrow edema-like lesion of the medial condyle (asterisk). Vertical tear is well delineated in both thin-slice 2D FS-PDWI and 3D FS-PD MPV image, but high signal intensity of the tear is more distinct in thin-slice 2D FS-PDWI. For bone marrow edema-like lesion of the medial condyle, thin-slice 2D FS-PDWI provides better image depiction than 3D FS-PD MPV image. 2D FS-PDWI indicates 2-dimensional fat saturated-proton density weighted image; 3D FS-PD MPV, 3-dimensional fat saturated-proton density multi planar voxel.

The supplemental table shows scores for abnormal findings for each structure in thin-slice 2D FS-PDWI and 3D FS-PD MPV image. The percentages of both scores of 5 (5/5) in the case with "abnormal findings" in thin 2D FS-PDWI and 3D FS-PD MPV image were 86% (74/86) and 67% (58/86), respectively (Table 5) and these showed a statistically significant difference (*p* < 0.05) (Figs. 4 and 5).

**Table 5:** Agreement with high confidence in “abnormal finding”.

Case	Location	Number of 5/5*		Number of image plane in location¶
		2D†	3D	
1	Medial meniscus	2	2	3
	Tibiofemoral joints	2	0	2
2	Femur	3	3	3
	Medial meniscus	3	3	3
	Tibiofemoral joints	2	2	2
	Femoropatellar joints	2	2	2
	Anterior cruciate ligament	0	1	3
3	Medial meniscus	3	3	3
	Tibiofemoral joints	2	2	2
4	Femoropatellar joints	2	2	2
5	Medial meniscus	0	2	3
6	Medial meniscus	3	3	3
	Tibiofemoral joints	2	2	2
	Femoropatellar joints	2	2	2
7	Femur	3	0	3
	Medial meniscus	3	3	3
	Tibiofemoral joints	2	2	2
	Anterior cruciate ligament	3	3	3
8	Femur	3	3	3
	Medial meniscus	3	3	3
	Tibiofemoral joints	2	2	2
9	Femur	3	0	3
	Medial meniscus	2	0	3
	Tibiofemoral joints	2	2	2
	Femoropatellar joints	2	2	2
	Anterior cruciate ligament	3	1	3
10	Femur	3	0	3
	Medial meniscus	2	1	3
	Tibiofemoral joints	2	2	2
11	Femur	3	0	3
	Medial meniscus	3	3	3
	Tibiofemoral joints	2	2	2
	Anterior cruciate ligament	0	0	3
Total		74	58	86

"Abnormal findings" were defined as "on both thin-slice 2D FS-PDWI and 3D FS-PD MPV images", " on two or more image planes (coronal image only for medial and lateral collateral ligament)", and “scored 4 or higher by two readers”.

*5/5 indicates that two readers gave a score of 5.

^¶^This represents the number of image plane defined to evaluate the abnormal findings of each location, as described in *Qualitative image evaluation methods*.

^†^*p* value < 0.05 by the chi-squared test.

## Discussion

It would have been impossible to acquire thin-slice 2D FS-PDWI with fine MPR images in a clinically acceptable scan time. However, we inferred that thin-slice 2D FS-PDWI that can fulfill the requirement of ideal image quality would be possible by the improvement of SNR with the application of dDLR. Thin-slice 2D FS-PDWI was compared with 3D FS-PDWI, which is now performed routinely. Thin-slice 2D FS-PDWI was scanned in coronal plane as the original image, from which MPR images were created. By contrast, 3D FS-PD MPV image was scanned in sagittal plane and MPR images were created. The reason for scanning thin-slice 2D FS-PDWI in coronal plane was to minimize the effects of the flow artifacts from the popliteal artery. By setting a high in-plane resolution to 0.5 × 0.5 mm, slice thickness to 1 mm, and gap to − 0.3 mm, we were able to create fine MPR images.

Many approaches were used to obtain the high-resolution images. Simply increasing the resolution would result in a decrease in SNR. Increasing the number of excitations would improve the SNR, but it would also increase the acquisition time and would not be acceptable for clinical practice^38^. Actually, dDLR uses multi-layered CNNs, which are trained by optimizing the CNN parameters to make the result of processing a low SNR image with CNNs closer to the high SNR image as a teacher image^9,10^. The decrease of SNR was anticipated due to decreased voxel volume and effects of a cross talk artifact by overlapping the gap^39^. This problem could be resolved by dDLR. We demonstrated that resolution by significantly lower CV in quantitative image evaluation, which is regarded as one indicator of image noise^40^, in thin-slice 2D FS-PDWI.

With image quality including edge sharpness, contrast resolution, fluid brightness, and uniformity of image, no significant difference were found between thin-slice 2D FS-PDWI and 3D FS-PD MPV image. One radiologist (Reader A) evaluated the sagittal and transverse images of edge sharpness in thin-slice 2D FS-PDWI as "non-acceptable" (Table 2), but these were MPR images of the same case. Coronal image was evaluated as "acceptable". In this case motion artifacts were strong. The evaluation was acceptable only on the original coronal image, but not on the reconstructed sagittal and transverse images. It is noteworthy that, in this case, both radiologists rated the motion artifact as "non-acceptable”. This result was derived from an accidental motion artifact created during the scanning of thin-slice 2D FS-PDWI. We expect that the same results as those obtained in the other cases would have been obtained if the patient had not moved. Depending on the method of k-space data sampling, motion artifacts are more likely to appear in the slice direction and in the phase direction in the 3D image compared to the 2D image^41,42^. Therefore, in thin-slice 2D image, the effects of motion artifacts on the original image to be scanned are slight and the effects on the MPR image can be expected to be small. In 3D image, however, both the original image and the MPR image are strongly affected. For flow artifacts, thin-slice 2D FS-PDWI were significantly worse than 3D FS-PD MPV image.

For the evaluation of anatomical structure visualization, no significant difference was found between thin-slice 2D FS-PDWI and 3D FS-PD MPV image. One radiologist (Reader A) evaluated sagittal image of femur, femoropatellar joints, ACL, and PCL as "non-acceptable" in thin-slice 2D FS-PDWI (Table 3). Actually, this represents the same case of motion artifacts as described above. Another noteworthy point is that significant flow artifacts observed in thin-slice 2D FS-PDWI did not negatively influenced the anatomical structure visualization when compared to 3D FS-PD MPV image.

Regarding inter-rater reliability coefficients in abnormal findings, thin-slice 2D FS-PDWI was found to have superiority of greater 3D FS-PD MPV image in overall Gwet’s AC_2_. In terms of the cartilage delineation of femoropatellar joints, 3D FS-PD MPV image was inferior to thin-slice 2D FS-PDWI. This might be attributable to the blurring effects of 3D images. Therefore, thin-slice 2D FS-PDWI is expected to indicate abnormal findings more constantly than 3D FS-PD MPV image does.

For the evaluation of abnormal findings for each anatomical structure, the percentages of both scores of 5 (5/5) was higher in thin-slice 2D FS-PDWI than in 3D FS-PD MPV image. Therefore, we can point out abnormalities with higher confidence in thin-slice 2D FS-PDWI than in 3D FS-PD MPV image. The case described above with motion artifact was not particularly bad for detecting abnormal findings. The higher consistency and confidence indicated that the abnormal signal can be depicted more clearly in thin-slice 2D FS-PDWI, in spite of flow artifact effects.

This study has several limitations. First, the number of patients included in the study was small. Future studies should examine data of more patients. Second, we evaluated only the image findings and did not compare them to the surgical findings. Further study examining the surgical findings must be conducted.

In conclusion, we were able to obtain thin-slice 2D FS-PDWI with high-resolution using dDLR and were able to create fine MPR images. This is the first of an attempt to make use of the high-resolution and high contrast of 2D image and treat it like a 3D volume. Because the acquisition time for thin-slice 2D FS-PDWI was shorter than that for 3D FS-PD MPV image and was within the acceptable range, it can be readily applicable to clinical practice. With regard to the evaluation of abnormal findings, we indicated findings on thin-slice 2D FS-PDWI with greater consistency and confidence. Thin-slice 2D FS-PDWI with dDLR would afford an ideal image. It can be considered as an alternative for 3D FS-PD MPV image for evaluating knee joint abnormality. Finally, because dDLR is adaptable to various sequences, it is expected to be applicable to other different joints.

## Supplementary Information

Supplementary Information.
