# Supplementary material for: Diagnostic advantage of thin slice 2D MRI and multiplanar reconstruction of the knee joint using deep learning based denoising approach
Source: Sci Rep. 2022 Jun 20;12:10362. doi: 10.1038/s41598-022-14190-1 (PMC9209466; doi:10.1038/s41598-022-14190-1)
Supplement: Supplementary file 1 — Supplementary Information. [file 41598_2022_14190_MOESM1_ESM.pdf]

Diagnostic advantage of thin slice 2D MRI and multiplanar reconstruction of the knee joint using deep learning based denoising approach

Takahide Kakigi, MD, PhD, Ryo Sakamoto, MD, PhD, Hiroshi Tagawa, MD, Shinichi Kuriyama, MD, PhD, Yoshihito Goto, MD, MPH, Masahito Nambu, Hajime Sagawa, Hitomi Numamoto, Kanae Kawai Miyake, MD, PhD, Tsuneo Saga, MD, PhD, Shuichi Matsuda, MD, PhD, Yuji Nakamoto, MD, PhD

Supplemental table: Score for abnormal findings for each structure in thin-slice 2D FS-PDWI and 3D FS-PD MPV image

**Thin-Slice 2D FS-PDWI**											
Score		5/5	5/4	4/4	4/3	3/3	3/2	2/2	2/1	1/1	Others
Femur	Coronal	8	1	0	1	0	0	0	0	2	5/1: 1
	Sagittal	7	1	1	1	0	0	0	0	2	5/1: 1
	Transverse	7	2	0	0	0	1	0	0	3	
Medial meniscus	Coronal	10	1	0	0	1	0	0	0	1	
	Sagittal	8	1	0	0	2	0	0	0	1	5/3: 1
	Transverse	8	2	0	0	1	0	0	0	1	5/3: 1
Lateral meniscus	Coronal	0	1	0	1	0	0	0	1	9	3/1: 1
	Sagittal	0	0	0	1	1	0	0	1	9	3/1: 1
	Transverse	0	1	0	0	1	0	0	0	10	3/1: 1
Articular cartilage (Tibiofemoral joints)	Coronal	11	0	0	0	0	0	0	1	1	
	Sagittal	11	0	0	0	0	0	0	1	1	
Articular cartilage (Femoropatellar joints)	Sagittal	8	2	2	0	0	0	0	0	1	
	Transverse	10	0	1	0	1	0	0	0	1	
Anterior cruciate ligament	Coronal	3	2	0	0	0	1	1	1	5	
	Sagittal	3	2	0	0	0	2	0	1	5	
	Transverse	3	2	0	0	0	2	0	1	5	
Posterior cruciate ligament	Coronal	0	0	0	0	0	0	0	2	11	
	Sagittal	0	0	0	0	0	0	1	1	11	
	Transverse	0	0	0	0	0	0	0	1	12	
Medial collateral ligament	Coronal	0	2	0	0	0	0	2	3	4	3/1: 2
Lateral collateral ligament	Coronal	0	0	0	0	0	0	1	3	9	

**3D FS-PD MPV Image**											
Score		5/5	5/4	4/4	4/3	3/3	3/2	2/2	2/1	1/1	Others
Femur	Coronal	2	3	1	0	0	0	0	0	6	4/1: 1
	Sagittal	2	3	1	0	0	0	0	1	5	4/1: 1
	Transverse	2	2	1	0	0	0	0	0	7	5/3: 1
Medial meniscus	Coronal	8	3	0	0	1	0	0	0	1	
	Sagittal	7	1	0	0	1	0	0	0	1	5/3: 3
	Transverse	8	2	0	0	1	0	0	0	1	5/3: 1
Lateral meniscus	Coronal	0	0	0	0	2	0	0	1	9	3/1: 1
	Sagittal	0	0	0	0	1	0	0	1	8	3/1: 3
	Transverse	0	0	0	0	1	1	0	0	9	3/1: 2
Articular cartilage (Tibiofemoral joints)	Coronal	8	2	0	0	0	0	0	2	1	
	Sagittal	8	1	0	0	0	0	0	2	1	5/3: 1
Articular cartilage (Femoropatellar joints)	Sagittal	4	0	0	1	0	0	0	2	1	5/3: 3, 3/1: 1, 4/1: 1
	Transverse	5	0	0	0	0	0	0	2	1	5/3: 3, 3/1: 1, 4/1: 1
Anterior cruciate ligament	Coronal	2	2	0	1	0	0	1	2	5	
	Sagittal	2	2	1	0	0	0	1	3	4	
	Transverse	1	3	0	1	0	0	1	2	5	
Posterior cruciate ligament	Coronal	0	0	0	0	0	1	0	4	8	
	Sagittal	0	0	0	0	0	0	1	4	8	
	Transverse	0	0	0	0	0	1	1	4	7	
Medial collateral ligament	Coronal	0	0	0	0	0	3	0	3	6	3/1: 1
Lateral collateral ligament	Coronal	0	0	0	1	0	0	0	2	10	

2D FS-PDWI indicates 2-dimensional fat saturated-proton density weighted image; 3D FS-PD MPV, 3-dimensional fat saturated-proton density multi planar voxel.
